# Identification of potential novel drug resistance mechanisms by genomic and transcriptomic profiling of colon cancer cells with p53 deletion

**DOI:** 10.1007/s00204-021-02979-4

**Published:** 2021-01-30

**Authors:** Onat Kadioglu, Mohamed Saeed, Nuha Mahmoud, Shaymaa Azawi, Kristin Mrasek, Thomas Liehr, Thomas Efferth

**Affiliations:** 1 Department of Pharmaceutical Biology, Institute of Pharmaceutical and Biomedical Sciences, Johannes Gutenberg University, Mainz, Germany; 2 Institute of Human Genetics, Jena University Hospital, Friedrich Schiller University, Jena, Germany

**Keywords:** Cancer, Chromosomal aberrations, Drug resistance, Genomic instability, Loss-of-function, Transcriptomics, Tumor suppressor

## Abstract

TP53 (p53) is a pivotal player in tumor suppression with fifty percent of all invasive tumors displaying mutations in the *TP53* gene. In the present study, we characterized colon cancer cells (HCT116 *p53* ^*−/−*^) with *TP53* deletion, a sub-line derived from HCT116-*p53* ^+*/*+^ cells. RNA sequencing and network analyses were performed to identify novel drug resistance mechanisms. Chromosomal aberrations were identified by multicolor fluorescence in situ hybridization (mFISH) and array comparative genomic hybridization (aCGH). Numerous genes were overexpressed in HCT116 *p53* ^*−/−*^ cells: *RND3/RhoE* (235.6-fold up-regulated), *DCLK1* (60.2-fold up-regulated), *LBH* (31.9-fold up-regulated), *MYB* (28.9-fold up-regulated), *TACSTD2* (110.1-fold down-regulated), *NRIP1* (81.5-fold down-regulated) and *HLA-DMB* (69.7-fold down-regulated) are among the identified genes with potential influence on multidrug resistance (MDR) and they are associated with cancer progression and tumorigenesis, according to previously published studies. Probably due to *TP53* deletion, disturbances in DNA repair and apoptosis are leading to aberrancies in cellular and organismal organization, ultimately increasing tumorigenesis and cancer progression potential. With NFκB, PI3K and HSP70, being at the center of merged protein network, and TH1-2 pathways, being among the influenced pathways, it can be speculated that the inflammatory pathway contributes to a resistance phenotype together with cell cycle regulation and heat-shock response. HCT116-*p53* ^*−/−*^ cells have more chromosomal aberrations, gains and losses in copy numbers than HCT116-*p53* ^+*/*+^ cells. In conclusion, numerous genomic aberrations, which might be associated with yet unknown drug resistance mechanisms, were identified. This may have important implications for future treatment strategies.

**Supplementary Information:**

The online version contains supplementary material available at 10.1007/s00204-021-02979-4.

## Introduction

*TP53* has been described as the guardian of the genome (Lane 1992). Upon detrimental damage caused by xenobiotic and carcinogenic substances, p53 maintains cellular integrity. Aberrations and damage in DNA are recognized by p53, leading to cell cycle arrest and DNA repair. In case of persistent damage exceeding the limit of cellular repair capability, p53 can trigger apoptosis. The mechanisms of apoptosis induced by p53 consist of transcriptional activation of *FAS*, *KILLER/DR5*, and the mitochondrial pathways (Green and Kroemer 2009). Furthermore, genes promoting cell survival such as *BCL2, IGFR, MCL-1*, survivin and *PIK3CA* are inhibited by p53 (Oren 2003; Riley et al. 2008). P53 plays role in various downstream processes, in addition to apoptosis and growth arrest, after activation by post-translational modifications such as phosphorylation, acetylation, and methylation (Bode and Dong 2004). It functions as a transcription factor responsible for maintaining the genomic integrity by regulating cell cycle arrest, DNA repair, and/or apoptosis-related pathways. In terms of cell cycle regulation, p53 activates p21/WAF1, an inhibitor for G2/M-specific cell division control protein 2 kinase and cyclin-dependent G1 kinase, subsequently leading to G2/M and G1 checkpoint control. Failure in arresting cells at both G1 and G2/M checkpoints due to mutated p53 can lead to drug resistance (Agarwal et al. 1995; Piovesan et al. 1998).

Both DNA repair and apoptosis mechanisms are essential to maintain a healthy condition in human cells. Under normal conditions, cells with excessive DNA damage or other aberrations are eliminated by apoptosis. If this control mechanism initiated by p53 is disrupted, abnormal cell proliferation with excessive DNA damage occurs. p53 mutations are among the main reason for disrupted DNA repair and apoptosis, which may initiate tumorigenesis due to increased population of abnormal cells which are more prone to mutations and chromosomally unstable. Abnormal proliferation of those cells with accumulated DNA damage because of nonfunctional p53 also affects the subsequent generations of cells with additional mutations. Ultimately, this leads to an increased risk of carcinogenesis.

P53 is mutated in more than 50% of all human carcinomas, and colorectal cancer is among the cancer types with frequent deleterious p53 mutations (Baker et al. 1989). Most of the mutations occur in the DNA-binding domain and lead either to protein-misfolding or disruption of the DNA-binding ability (Hainaut et al. 1997). The loss of its apoptotic function is an important reason for the development of radio- and drug-resistant cancer cells (Bertheau et al. 2008; Chen et al. 2012). Moreover, tumors with p53 mutations are commonly characterized by aggravated metastasis and genomic instability (Liu et al. 2010; Muller and Vousden 2013). Additional oncogenic functions of mutant p53 include promoting invasion, migration, angiogenesis and proliferation, which can lead to enhanced drug resistance and mitogenic defects (Muller and Vousden 2013). The above functions are just a few of the plethora of multiple pathways by which mutant p53 governs cancer progression (Muller and Vousden 2013). For instance, p53 has an impact also on drug metabolism (Krais et al. 2016; Wohak et al. 2018) and cell metabolism by limiting glycolysis and facilitating mitochondrial respiration (Gomes et al. 2018; Matsuura et al. 2016).

Resistance to multiple drugs has been well studied in ATP-binding cassette (ABC) transporters, which mediate the multidrug resistance (MDR) phenotype. Multiple drug resistance is, however not restricted to ABC transporters and other MDR phenomena have also been described, including, p53, Bcl-2, the proliferation rate of tumors and others (Efferth et al. 2008; Hientz et al. 2017; Reed 1995). Microarray analyses were previously performed for HCT116 cell line (Bhattacharjee et al. 2005; Kabir et al. 2018; Khonthun et al. 2020; Ma et al. 2017), but the application of genomics and transcriptomics methods to isogenic knockout cells allows a superior and deeper comparison between cell lines to identify novel drug resistance mechanisms.

In this study, we applied RNA sequencing, array comparative genomic hybridization (aCGH) and multicolor fluorescence in situ hybridization (mFISH) to analyze HCT116 *p53* ^+*/*+^ colon cancer cells and its drug-resistant subline with *p53* deletion, HCT116 *p53* ^*−/−*^, to characterize genes, pathways, protein networks and chromosomal aberrations responsible for drug resistance in the HCT116 *p53* ^*−/−*^ cell line. Overall, this study shall provide a better overview of the full complexity of mechanisms and genetic alterations in colon cancer cells and their contribution to drug resistance that occurred upon *p53* deletion.

## Materials and methods

### Cell culture

HCT116 *p53*^+*/*+^ and its drug-resistant HCT116 *p53* ^*−/−*^ subline, which were generously provided by Dr. B. Vogelstein and H. Hermeking (Howard Hughes Medical Institute, Baltimore, MD, USA) (Bunz et al. 1998) were grown as described previously (Saeed et al. 2015). HCT116 *p53* ^*−/−*^ cells possess a significant mitotic checkpoint deficit such that they cannot respond normally to DNA-damaging agents, enter mitosis and subsequently replicate their genomes in the presence of DNA damage (Bunz et al. 1998). The drug resistance profile of HCT116 *p53* ^*−/−*^ has been studied during the past years. Compared to wild-type cells, these knockout cells reveal resistance to established anticancer drugs of diverse pharmacological classes (doxorubicin, 5-fluorouracil and 5′-deoxy-5-fluorouridine, cisplatin and oxaliplatin, etoposide, and vincristine) as well as to investigational cytotoxic compounds with activity against cancer (arsenic trioxide as PML/RARA inhibitor, nutlin-3a as *p53* activator, the fluoropyrimidine F10, the HDAC inhibitor entinostat and the synthetic polyamine DENSpm) and even cytotoxic but non-cancer drugs (the antimalarial quinacrine, the anticonvulsant valproic acid and the anti-inflammatory and COX1/2-inhibitory ibuprofen (Brachtendorf et al. 2018; Bunz et al. 1999; Coker-Gurkan et al. 2015; Dawood et al. 2018; Dominijanni and Gmeiner 2018; Gunasegaran et al. 2020; Hernlund et al. 2008; Janssen et al. 2008; Kralova et al. 2009; Lin et al. 2004; Mohapatra et al. 2012; Sonnemann et al. 2014; Terranova-Barberio et al. 2017).

### RNA sequencing

The procedure was previously described (Kadioglu et al. 2016a). Gene expressions were quantified using the FPKM (fragments per kilobase of transcript per million mapped reads) measure (Choudhri et al. 2018; Wesolowski et al. 2013). The deregulation of genes in HCT116 *p53* ^*−/−*^ cells was calculated by dividing overall FPKM values of genes in HCT116 *p53* ^*−/−*^ cells by those in HCT116 *p53* ^+*/*+^ cells.

### Pathway and network analysis

Fold change in RNA expression of ± 7 were applied for filtering (Kadioglu et al. 2016a), and then the deregulated gene list was subjected to Ingenuity Pathway Analysis (IPA) (QIAGEN Redwood City, USA, www.qiagen.com/ingenuity) to identify specific networks and pathways in HCT116 *p53* ^*−/−*^ cells.

### mFISH

HCT116 *p53* ^*−/−*^ and HCT116 *p53* ^+*/*+^ cells were cytogenetically prepared to obtain metaphase spreads according to standard procedures and analyzed using molecular cytogenetics. mFISH was performed as previously reported using human whole chromosome paints as probes (Kadioglu et al. 2016a; Liehr et al. 2009a, b; Liehr and Pellestor 2009).

### aCGH

Whole genomic DNA was extracted from HCT116 *p53* ^*−/−*^ and HCT116 *p53* ^+*/*+^ cells with QIAmp DNA mini kit (QIAGEN GmbH, Hilden, Germany). aCGH was performed as previously reported (Aust et al. 2013).

### Western blotting

The protein expression levels of selected genes (i.e., ANGPT2 and catalase) were evaluated in HCT116 *p53* ^*−/−*^ and HCT116 *p53* ^+*/*+^ cells to validate their deregulation found by RNA sequencing analysis as previously described (Kadioglu et al. 2016a). Briefly, total proteins were extracted from cells using protein extraction buffer (M-PER™ mammalian protein extraction reagent mixed with 1% Halt™ protease inhibitor cocktail, Thermo Fisher Scientific). Samples equivalent to 30 µg were loaded to 10% SDS-PAGE to be separated and then transferred to Ruti®-PVDF membranes (Millipore, Billerica, MA, USA). The membranes were blocked with 5% BSA (Carl Roth, Karlsruhe, Germany) for 1 h and probed with the selected primary antibodies at 4 °C against ANGPT2, catalase and β-actin (for all 1:1000, Cell Signaling Technology, Frankfurt, Germany). After 24 h, the membranes were incubated with secondary antibody conjugated to HRP (1:2000, Cell Signaling Technology) for 1 h and detected with Luminata™ Classico Western HRP substrate (Merck Millipore Darmstadt, Germany). Images were analyzed using ImageJ software (NIH, Bethesda, MD, USA).

## Results

### *Differential gene expression profile of HCT116 p53*^*−/−*^* cells, downstream pathways and network analysis*

Ratios of RNA-seq-derived FPKM values for the expression of each gene in HCT116 *p53* ^*−/−*^ cells were considered as fold change of gene expression in comparison to that of HCT116 *p53* ^+*/*+^ cells. For further analysis, differential gene expression with a threshold of ± 7 was taken into account (Kadioglu et al. 2016a), which yielded 300 differentially expressed genes (Supplementary Table 1). The top 10 up- and down-regulated genes in HCT116-*p53* ^*−/−*^ cells are listed in Table 1. *RND3* (+ 235.6), *MCPH1* (+ 85.7) and *MYB* (+ 28.9) were among the most up-regulated genes, whereas *DPEP1* (− 431.9), *ICAM1* (− 166.5) and *NPM2* (− 85.4) were the most down-regulated genes.

**Table 1:** Top 10 up- and down-regulated genes in HCT116 *p53 *^*−/−*^ cells compared to HCT116 *p53* ^+*/*+^ cells

Gene	Differential expression (fold change)
Upregulation	
* RND3*	235.633
* MCPH1*	85.701
* DCLK1*	60.193
* ZNF772*	37.039
* ANKRD31*	37.028
* ZNF419*	32.420
* LBH*	31.954
* MYB*	28.855
* MIR4477B*	26.768
* MST1*	25.177
Down-regulation	
* DPEP1*	− 431.858
* ICAM1*	− 166.463
* EHF*	− 163.282
* TACSTD2*	− 110.073
* LAMA4*	− 104.721
* NPM2*	− 85.415
* NRIP1*	− 81.519
* HLA-DMB*	− 69.678
* HCG4B*	− 61.255
* MIR564, TMEM42*	− 58.380

**Fig. 1:**
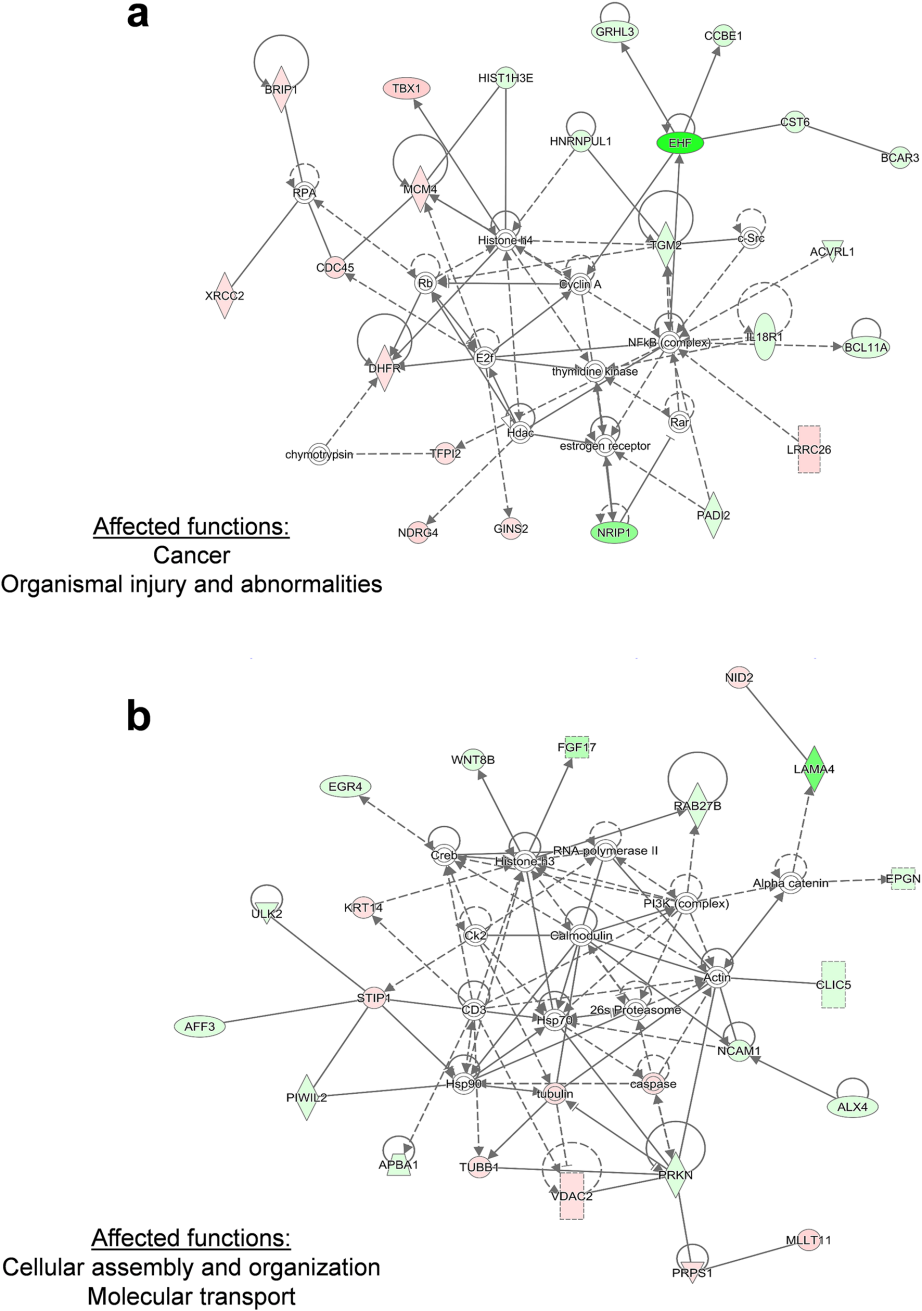

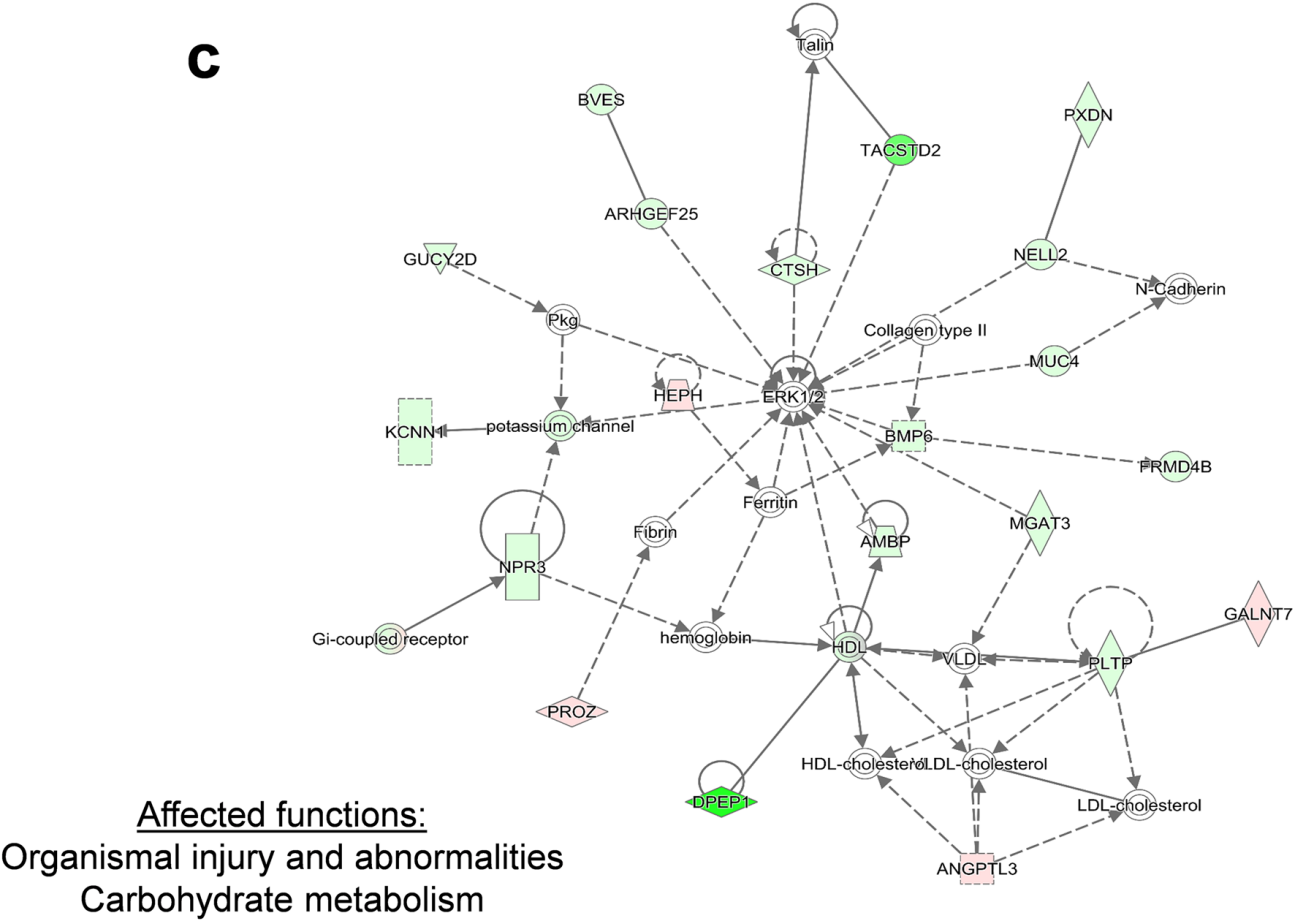
Affected protein networks in HCT116 *p53 *^*−/−*^ cells in comparison to HCT116 *p53* ^+*/*+^ cells. Genes that are labelled in green were down-regulated, and genes that are labelled in red were up-regulated. The top three networks were depicted. **a** Network 1 **b** Network 2 **c** Network 3

In network 1, histone H4, cyclin A and NFκB possessed the highest number of nodes, CD3 and Hsp70 had the highest number of nodes in the center of network 2. “Cancer”, “organismal injury and abnormalities” in network 1, whereas “cellular assembly and organization” and “molecular transport” in network 2 were the affected biological functions. Erk1/2 showed the highest number of nodes in network 3. “Organismal injury and abnormalities” and “carbohydrate metabolism” were the affected biological functions in network 3 (Fig. 1).

Several genes known to be involved in drug resistance were deregulated, implying that HCT116 *p53* ^*−/−*^ cells exerted a multi-factorial resistance phenotype. If a fold change threshold of ± 7.0 was applied, one DNA repair gene, one oxidative stress gene, and one transcription factor gene were among the deregulated resistance genes implying that genes from those gene classes may have an important influence on the MDR phenotype of HCT116 *p53* ^*−/−*^. These genes are depicted in Table 2 and a full list of all deregulated genes involved in resistance mechanisms is provided in Supplementary Table 2.

**Table 2:** Deregulated genes involved in classical drug resistance mechanisms in HCT116 *p53 *^*−/−*^ cells compared to wild-type HCT116 *p53* ^+*/*+^ cells (threshold: ± sevenfold changed expression)

	Differential expression (fold change)
DNA repair	
* XRCC2*	11.200
* BRIP1*	7.711
Oxidative stress	
* NCF2*	15.131
* MB*	− 8.093
Transcription factors	
* MYB*	28.855
* NFATC4*	− 7.619

**Fig.2:**
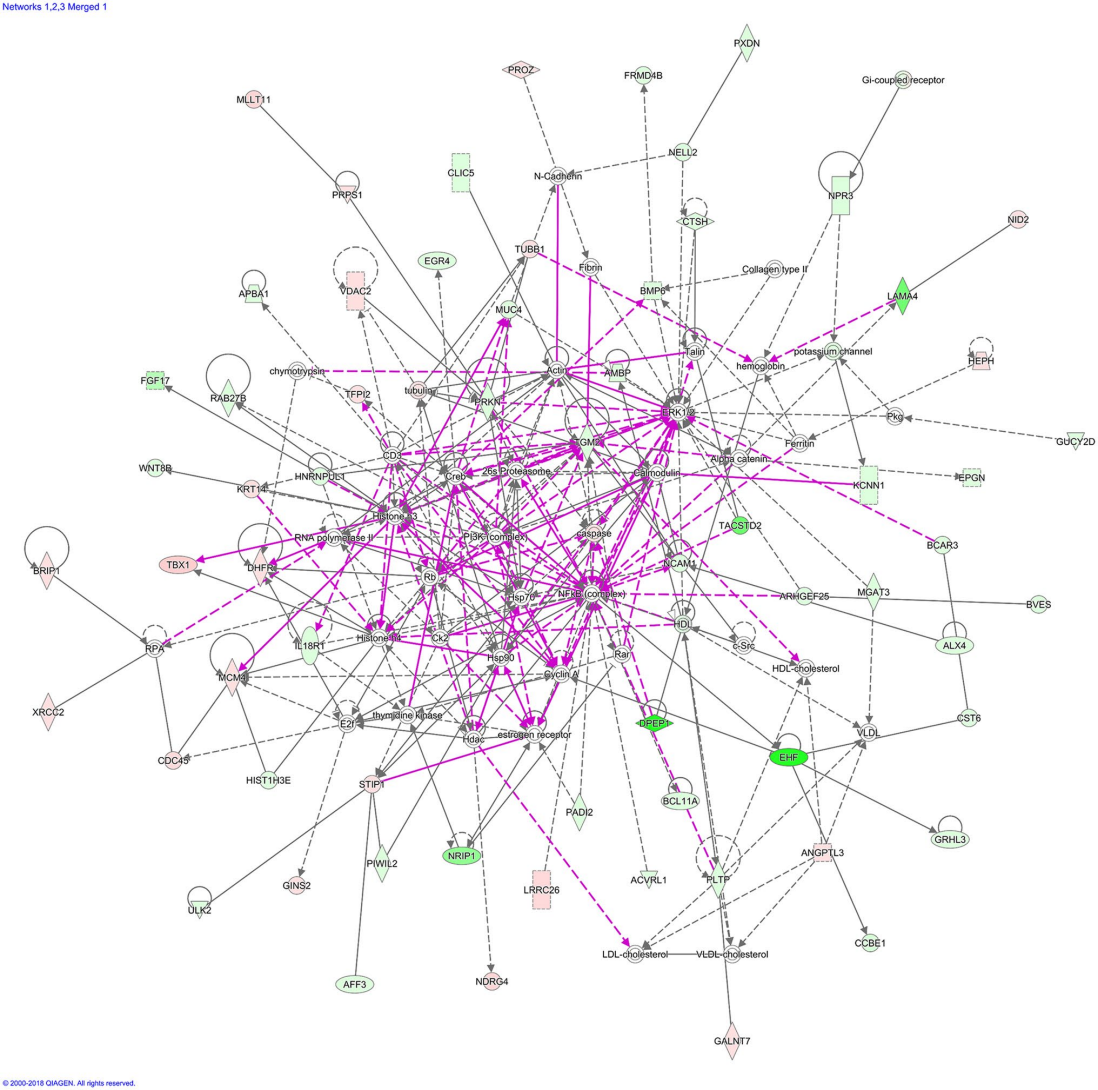
Merged protein network of network 1, 2, and 3

The top three networks were merged and the merged network was further analyzed. As can be seen from Fig. 2, NFκB resided in the center of the merged network together with PI3K and HSP70.

**Fig.3:**
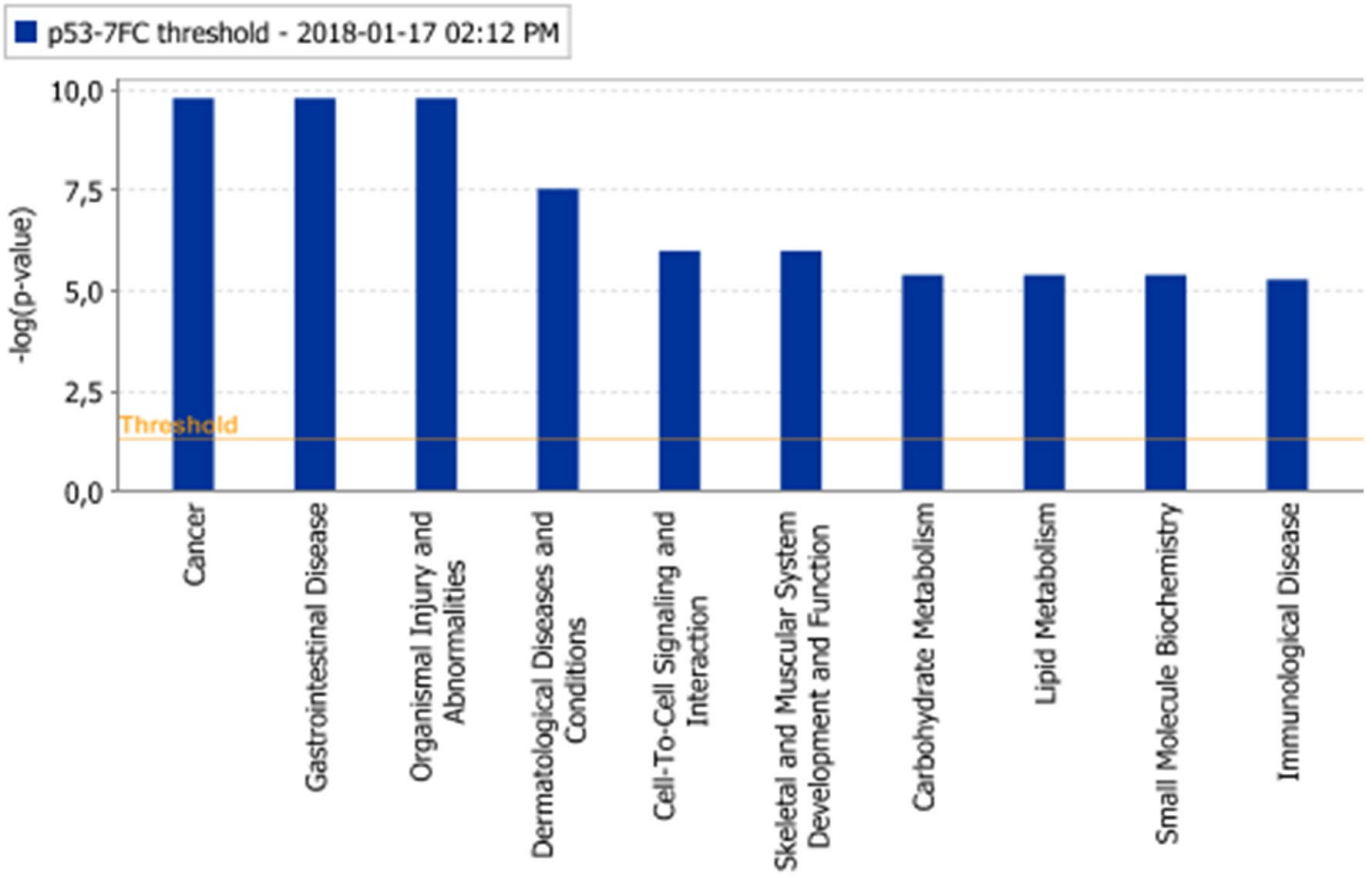
Affected biological functions (Top 10) in HCT116 *p53 *^*−/−*^ cells in comparison to HCT116 *p53* ^+*/*+^ cells. The orange line depicts the statistical significance threshold (*p* = 0.05)

“Cancer”, “organismal injury and abnormalities” and “cell to cell signaling and interaction” were among the most affected biological functions in HCT116 *p53* ^*−/−*^ cells (Fig. 3). Genes residing at the top 10 biological function list are shown in Supplementary Table 3.

**Table 3:** Deregulated *p53* target genes in HCT116 *p53 *^*−/−*^ cells compared to HCT116-*p53* ^+*/*+^ cells

Gene	Differential expression (fold change)
Upregulation	
* SPATA18*	4.207
* CSF1*	4.139
* TSPAN11*	3.365
* TP53I3*	3.098
* PTP4A1*	2.352
* ABCA12*	2.058
* ZNF79*	2.032
* DUSP14*	2.026
* FAM210B*	1.975
* RPS27L*	1.969
* GDF15*	1.856
* NADSYN1*	1.692
* FAS*	1.639
* FAM212B*	1.611
* ATF3*	1.599
* HSPA4L*	1.579
* TRIAP1*	1.549
* AEN*	1.547
* MDM2*	1.513
Down-regulation	
* GRHL3*	− 7.153
* PRDM1*	− 5.224
* PADI4*	− 5.164
* CPE*	− 4.718
* FAM198B*	− 3.568
* WDR63*	− 3.419
* FUCA1*	− 2.999
* CDIP1*	− 2.582
* ASTN2*	− 2.441
* SULF2*	− 2.315
* TNFRSF10D*	− 2.205
* TNFRSF10B*	− 2.058
* KITLG*	− 2.047
* PGF*	− 2.047
* BAX*	− 2.018
* ACER2*	− 2.017
* CCNG1*	− 1.874
* GPR87*	− 1.873
* XPC*	− 1.867
* SERTAD1*	− 1.863
* ARHGEF3*	− 1.834
* SYTL1*	− 1.804
* CD82*	− 1.789
* FDXR*	− 1.756
* EPS8L2*	− 1.718
* SESN1*	− 1.706
* SESN2*	− 1.654
* APOBEC3C*	− 1.648
* TP53INP1*	− 1.609
* DYRK3*	− 1.586
* ANKRA2*	− 1.581
* ORAI3*	− 1.558
* CES2*	− 1.535

**Fig. 4:**
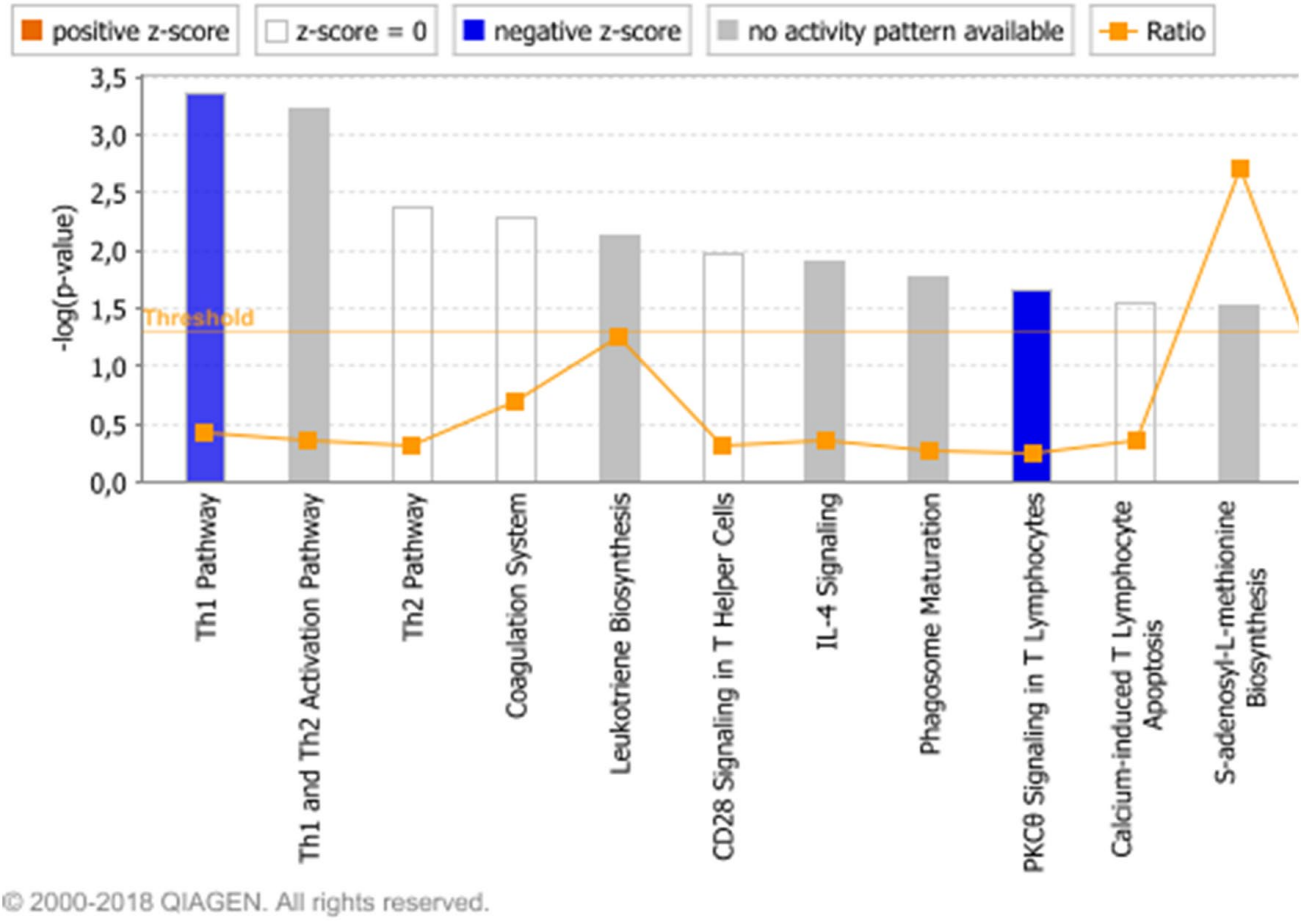
Affected signalling pathways (Top 10) in HCT116 *p53 *^*−/−*^ cells in comparison to HCT116 *p53* ^+*/*+^ cells. The orange line depicts the statistical significance threshold (*p* = 0.05) and the orange chart depicts the ratio of deregulated genes in each pathway

“Th1 pathway” (*p* value: 0.000437), “IL4 signaling” (*p* value: 0.012589) were among the most significant signaling pathways in HCT116 *p53* ^*−/−*^ cells implying the possible immune response pathways influence on drug resistance (Fig. 4).

Among the 116 *p53* target genes (Fischer 2017), 33 were down-regulated and 19 were up-regulated (fold changes were above a threshold of ± 1.5) as can be seen in Table 3.

**Fig. 5:**
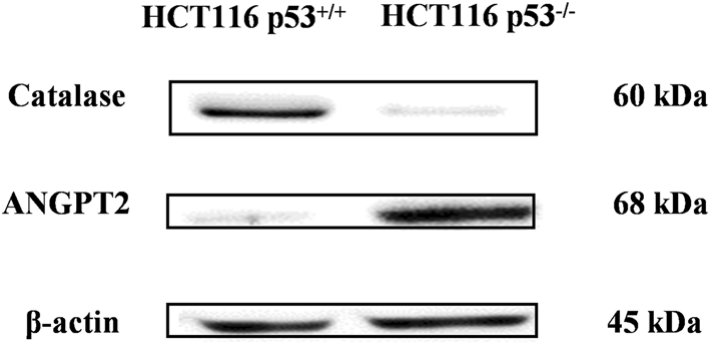
Protein expression of ANGPT2 and catalase in HCT116 *p53 *^*−/−*^ and HCT116 *p53* ^+*/*+^ cells as determined by Western blotting

A validation of the selected genes was performed at the protein level for ANGPT2 and catalase. As shown in Fig. 5, ANGPT2 was up-regulated (+ 8.7-fold), whereas catalase was down-regulated (− 1.9-fold) in HCT116 *p53* ^*−/−*^ cells, correlating with the RNA sequencing output and validating the RNA expression data at the protein level.

### mFISH

**Fig. 6:**
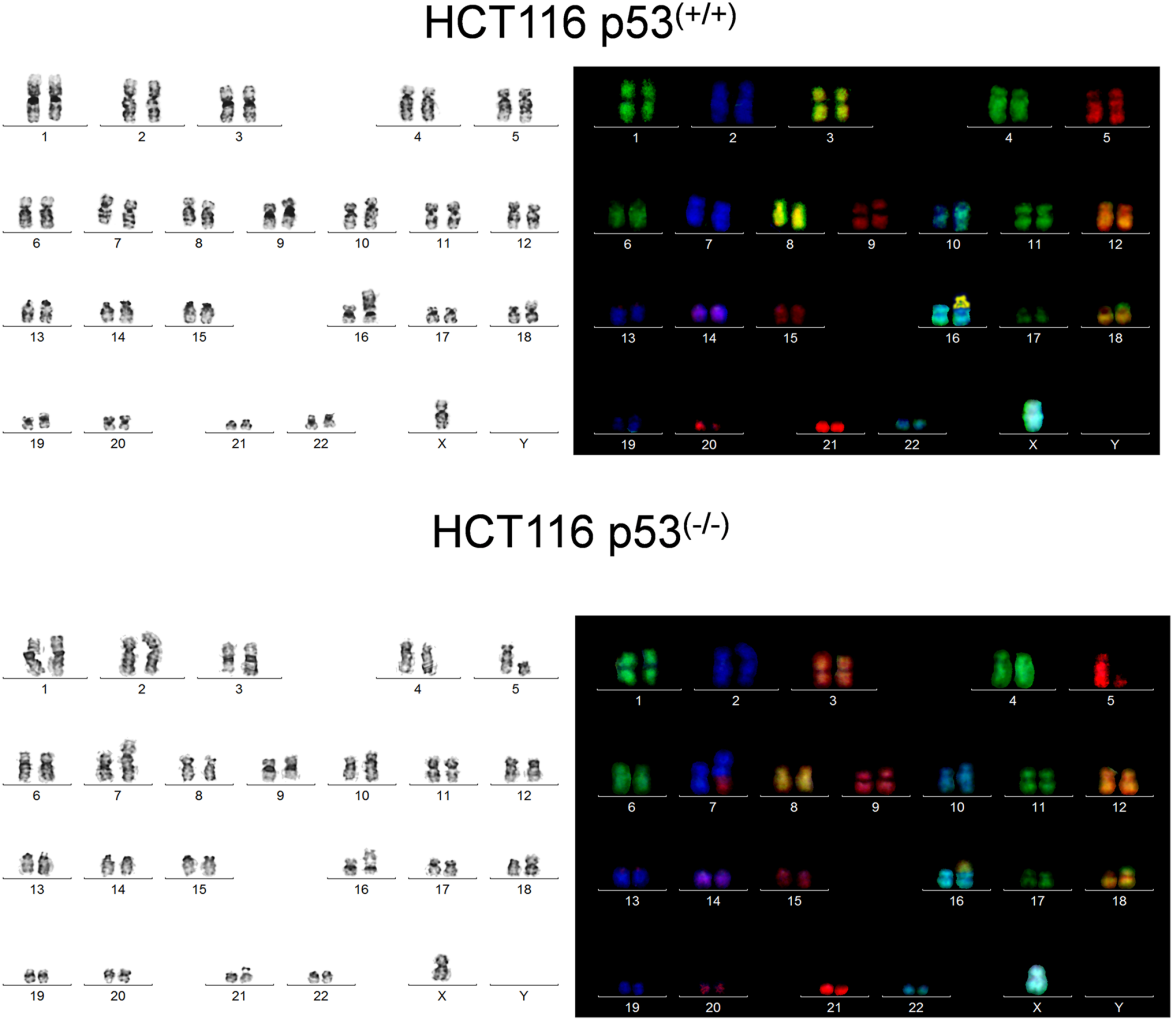
mFISH analysis of HCT116 *p53* ^+*/*+^ (**a**) and HCT116 *p53 *^*−/−*^ (**b**) cells

HCT116-*p53* ^+*/*+^ cells showed the karyotype 45 < 2n > , X, dup(10)(q?q?), der(16)t(8;16)(p13;?), der(18)t(17;18)(?q;p11.2), whereas HCT116-*p53* ^*−/−*^ cells had 45 < 2n > X, t(5;7)(q1?3;p22), dup(10)(q?q?), der(16)t(8;16)(p13;?), der(18)t(17;18)(?q;p11.2). The results of the mFISH analyses are depicted in Fig. 6. HCT116-*p53* ^*−/−*^ cells had a clonal de novo balanced translocation t(5;7)(q1?3;p22) compared to HCT116-*p53* ^+*/*+^ cells.

### aCGH

*HCT116 p53* ^+*/*+^: Chromosomal amplification and deletions were well reflected in the deregulation of gene expression as observed in RNA Seq analysis. *LVRN* was 3.3-fold down-regulated and *AP3S1* was 1.7-fold up-regulated. The corresponding chromosomal locus (5q23.1) was amplified. *FLJ42393* was 1.7-fold down-regulated. There was a deletion at the corresponding chromosomal locus (3q27.3–q28). The results are summarized in Table 4.

**Table 4:** Chromosomal aberrations and corresponding deregulated genes. Comparison between aCGH and RNA sequencing profiles

Chromosomal locus	Cytoband	# Probes	Amp/Del	Gene
HCT116 *p53* ^+*/*+^				
chr3:187898258-188,080,406	q27.3–q28	15	− 1.088643	*FLJ42393*
chr5:115220574-115433864	q23.1	19	0.672502	*AP3S1*
				*LVRN*
HCT116 *p53 *^*−/−*^				
chr2:139060642-139586488	q22.1	30	0.576345	*NXPH2*
chr3:98146718-98600450	q11.2–q12.1	33	0.480192	*OR5K2*
chr6:162343673-162707662	q26	32	− 0.797749	*PARK2*

*HCT116 p53* ^*−/−*^: Compared to HCT116 *p53* ^+*/*+^ cells, more amplifications and deletions were observed in HCT116-*p53* ^*−/−*^ cells. This implies that *p53* deletion led to an accumulation of additional chromosomal aberrations, amplifications and deletions. *NXPH2* was 1.7-fold up-regulated, and there was an amplification at the corresponding chromosomal locus. *OR5K2* was 1.7-fold up-regulated, and there was an amplification at the corresponding chromosomal locus. *PARK2* was 8.1-fold down-regulated, and there was a deletion at the corresponding chromosomal locus. Correlation of aCGH data with RNA-Seq results clearly showed a differential expression of genes at the corresponding chromosomal locus amplification/deletion, as can be seen in Table 4. Overall aCGH results are depicted in Table 5.

**Table 5:** Overall aCGH results

Chromosome	Cytoband	#Probes	Amp/Del	*P* value	Annotations
HCT116 *p53* ^+*/*+^					
chr3:114336335-114441291	q13.31	10	0.930778	1.43E-11	*ZBTB20, CNV_98410*
chr3:187898258-188080406	q27.3–q28	15	− 1.088643	7.50E-22	*LPP, FLJ42393, CNV_8438…*
chr4:169510103-169718417	q32.3	18	− 1.000585	3.99E-22	*PALLD, CNV_68838, CNV_6350…*
chr5:115220574-115433864	q23.1	19	0.672502	1.72E-11	*AP3S1, LVRN, COMMD10*
chrX:67606357-67674714	q12	7	1.187426	6.11E-13	*OPHN1*
chrY:6,689,115-6,823,761	p11.2	12	− 4.324700	1.07E-10	*AMELY, TBL1Y*
HCT116 *p53 *^*−/−*^					
chr2:139060642-139586488	q22.1	30	0.576345	4.63E-14	*SPOPL, NXPH2, CNV_6001…*
chr3:98146718-98600450	q11.2–q12.1	33	0.480192	3.77E-11	*OR5K1, OR5K2, CLDND1…*
chr3:116781727-117480699	q13.31–q13.32	18	− 0.840402	1.93E-17	
chr3:188,915,318–189,233,666	q28	16	0.678746	7.43E-11	*TPRG1, CNV_6224, CNV_36118…*
chr6:162343673-162707662	q26	32	− 0.797749	8.35E-27	*PARK2, CNV_3649, CNV_8532…*
chr12:19212202-19371177	p12.3	13	− 0.764834	3.82E-11	*PLEKHA5, CNV_113275, CNV_5296…*
chr14:105957346-107258824	q32.33	85	0.333574	2.22E-13	*C14orf80, TMEM121, KIAA0125…*
chr15:94197624-94591102	q26.1—q26.2	23	0.741784	2.28E-17	*CNV_47871, CNV_34628, CNV_9268…*
chr16:78,328,380–78,670,327	q23.1	24	− 1.124667	1.46E-37	*WWOX, CNV_4014, CNV_3128…*
chr20:14928568-15317311	p12.1	33	− 0.608444	7.87E-17	*MACROD2, CNV_9315, CNV_30119…*
chrX:29398858-29528742	p21.2	11	0.881627	2.58E-12	*IL1RAPL1, CNV_3265*
chrX:96427803-96727959	q21.33	25	− 3.569660	3.65E-319	*DIAPH2, CNV_68008*

Observed in both cell lines					
Chromosome	Cytoband	Annotations	Chromosome	Cytoband	Annotations
chr1:192899886-193202164	q31.2	*UCHL5, TROVE2, GLRX2…*	chr16:78949665-90163114	q23.1—q24.3	*WWOX, MAF, DYNLRB2…*
chr2:89427365-90242018	p11.2	*CNV_34427, CNV_35873, CNV_107994*	chr17:34450405-34475514	q12	*CNV_4031, CNV_8842, CNV_34507…*
chr2:141719777-142150170	q22.1	*LRP1B, CNV_98004, CNV_9962…*	chr17:43457048-81093254	q21.31—q25.3	*ARHGAP27, SH3D20, PLEKHM1…*
chr2:205437915-205794583	q33.3	*PARD3B, CNV_63286, CNV_3405…*	chr19:20644642-20984793	p12	*ZNF737, ZNF626, CNV_4070…*
chr3:60193460-60230828	p14.2	*FHIT, CNV_8983, CNV_51130…*	chr22:24347959-24390254	q11.23	*LOC391322, GSTT1, GSTTP2*
chr4:19271201-19814969	p15.31	*CNV_98645, CNV_91960, CNV_8998…*	chrX:550458-2687250	p22.33	*SHOX, CRLF2, CSF2RA…*
chr4:91321265-91603292	q22.1	*FAM190A, CNV_29746, CNV_98795…*	chrX:29548179-29946620	p21.2	*IL1RAPL1, CNV_3265, CNV_9860…*
chr4:184337598-185694854	q35.1	*CDKN2AIP, ING2, RWDD4A…*	chrX:154947952-155097214	q28	*SPRY3, CNV_0828, CNV_68067…*
chr5:104011349-104445773	q21.2	*RAB9P1, CNV_9021, CNV_51512…*	chrY:500458-2637250	p11.32–p11.31	*SHOX, CRLF2, CSF2RA…*
chr6:1797129-2265280	p25.3	*GMDS, CNV_51856, CNV_69297…*	chrY:2650450-9901314	p11.31–p11.2	*SRY, RPS4Y1, ZFY…*
chr6:32480027-32521929	p21.32	*HLA-DRB5, HLA-DRB6, CNV_3603…*	chrY:2808108-3163644	p11.31–p11.2	*ZFY*
chr7:133788914-133833057	q33	*LRGUK, CNV_3704, CNV_36620…*	chrY:3713948-5471518	p11.2	*PCDH11Y, CNV_31579, CNV_59553…*
chr8:39237438-39374789	p11.22	*ADAM5P, ADAM3A, CNV_2749…*	chrY:7117061-7159833	p11.2	*PRKY*
chr8:67392636-146294098	q13.1–q24.3	*C8orf46, MYBL1, VCPIP1…*	chrY:7382914-7663842	p11.2	*TTTY16, CNV_83913, CNV_97170…*
chr10:100050353-131197707	q24.2–q26.3	*PYROXD2, MIR1287, HPS1…*	chrY:13992304-28767604	q11.21–q11.23	*TTTY15, USP9Y, DDX3Y…*
chr10:131388943-135434178	q26.3	*MGMT, EBF3, GLRX3…*	chrY:14443070-14489146	q11.21	*CNV_0830, CNV_37072*
chr12:66303379-66937395	q14.3	*HMGA2, LLPH, TMBIM4…*	chrY:15192361-15251375	q11.221	
chr12:76107468-76705883	q21.2	*PHLDA1, NAP1L1, CNV_101564*	chrY:15704763-15956423	q11.221	*TMSB4Y*
chr14:22360671-22936054	q11.2	*CNV_34544, CNV_4828, CNV_61368…*	chrY:21718251-21782669	q11.222	*CYorf15A, CYorf15B, CNV_31588…*
chr16:106271-724192	p13.3	*SNRNP25, RHBDF1, MPG…*	chrY:22920321-23150221	q11.223	*RPS4Y2, CNV_31588*
chr16:21475039-21991860	p12.2	*LOC100271836, SLC7A5P2, METTL9…*	chrY:28485304-28722435	q11.23	*CNV_4185, CNV_31592*
chr16:78678935-78724670	q23.1	*WWOX*	chrY:59050958-59200220	q12	*SPRY3, CNV_83981, CNV_97244…*

## Discussion

In the present study, we aimed to identify novel drug resistance genes by using colon cancer cell line, HCT116 *p53* ^+*/*+^ and the drug-resistant HCT116 *p53* ^*−/−*^ subline with *TP53* deletion as a model. The gene expression profiles, affected signaling pathways, biological functions and chromosomal abnormalities were identified by RNA sequencing, mFISH and aCGH.

Several genes known to be involved in drug resistance were deregulated supporting a multi-factorial resistance phenotype in HCT116 *p53* ^*−/−*^ cells, those identified genes in various drug resistance clusters including apoptosis, DNA repair, ferroptosis, glutathione related, heat shock, oxidative stress, transcription factors as listed in Supplementary Table 2 may have an important influence on the MDR phenotype.

The most up-regulated gene *RND3/RhoE* (+ 235.6-fold) was previously associated with tumor invasion, metastasis and was reported as a potential marker of drug resistance of gastric cancer as well as relapse and prognosis for colorectal cancer cases (Chang et al. 2014; Li et al. 2009; Zhou et al. 2013). *CARD11* mutations have been associated with ibrutinib (Bartlett et al. 2018). *CARD11* (− 7.4-fold) appeared in the list of genes in the top five biological functions (Supplementary Table 3), indicating that apoptosis inhibition upon down-regulation of CARD11 might play an important role in the drug resistance phenotype of HCT116 *p53* ^*−/−*^ cells. It was reported that CARD11 contributes to ibrutinib resistance in cancer (Grommes et al. 2017; Wu et al. 2016) supporting our observation that CARD11 could play role in drug resistance phenotype of HCT116 *p53* ^*−/−*^ cells. *DCLK1* (+ 60.2-fold) has been reported to be associated with chemoresistance to cisplatin in non-small cell lung cancer cells and targeting *DCLK1* by miR539 led to increased sensitivity to cisplatin (Deng et al. 2018). *DCLK1* has also been associated with drug resistance in colorectal cancer, pancreatic cancer, and kidney cancer (Ge et al. 2018; Makino et al. 2020; Qu et al. 2019). *LBH* (+ 31.9-fold) has been reported as a potential marker for hepatocellular carcinoma, as its overexpression was associated with poor prognosis (Chen et al. 2018). *Myb* (+ 28.9-fold expression in knockout cells) is an oncogenic transcription factor playing a role in the promotion of leukemic cell transformation (Introna and Golay 1999). *Myb* was linked to cisplatin resistance in colon cancer cells (Funato et al. 2001). It is also involved in the development and progression of several solid tumors, including melanoma (Ramsay and Gonda 2008; Schultz et al. 2009). Loss of *TACSTD2* promoted squamous cell carcinoma progression and resistance through attenuating chemotherapeutic reagent-induced apoptosis, implying that *TACSTD2* could be used as a marker for pathological grading of SCC (Wang et al. 2014). Interestingly, it was 110.1-fold down-regulated in HCT116 *p53* ^*−/−*^ cells, pointing out that *TACSTD2* down-regulation could be a mechanism contributing to aggressive growth and MDR of HCT116 *p53* ^*−/−*^ cells. Migration and invasion of esophageal squamous cell carcinoma cells were enhanced upon *NRIP1* down-regulation (Ni et al. 2018). We observed that *NRIP1* was 81.5-fold down-regulated in HCT116 *p53* ^*−/−*^ cells, implying that *NRIP1* down-regulation could play a role in the MDR phenotype. *HLA-DMB* (− 69.7-fold) belongs to the major histocompatibility complex class II genes, and higher HLA-DMB expression was associated with higher survival rate via increased CD8 lymphocyte numbers in advanced-stage serous ovarian cancer (Callahan et al. 2008). Down-regulation of *HLA-DMB* may be linked with the MDR phenotype of HCT116 *p53* ^*−/−*^ cells by influencing tumor aggressiveness.

The *NCF2* gene (+ 15.1-fold expression in knockout cells) encodes a subunit of NOX2. Depletion of NOX2 subunits reduced the formation of lung metastases following intravenous injection of murine tumor cells (Martner et al. 2019). Up-regulation of *NCF2* promoted gastric cancer metastasis by LINC1410-miR-532-5p-NCF2-NF-κB feedback loop activation (Zhang et al. 2018).

Overexpression of the *MYB* transcription factor (+ 28.8-fold expression in knockout cells) has been associated with poor prognosis and was frequently observed in colorectal cancer (CRC) (Cross et al. 2015). Another study pointed out that *MYB* expression in tumor cells due to its tumorigenic role modulated the host immune response, which has the potential to influence the use of immunotherapy in CRC patients (Millen et al. 2016). *MYC* (+ 5.1-fold) is another transcription factor with a critical role in tumorigenesis. It regulates the expression of cell cycle related genes, and overexpression was observed in various cancer types, including colon cancer (Kadioglu et al. 2016b; Pelengaris et al. 2002). We have identified IL4 signaling among the most affected signaling pathways in HCT116 *p53* ^*−/−*^ cells implying that immune response pathways possibly influence drug resistance. One study stated that IL-4 can augment BCR-signalling and reduce the effectiveness of BCR-kinase inhibitors such as ibrutinib in CLL cells (Blunt et al. 2017). Another study reported that innate immune pathway activation via the interleukin-1 receptor-associated kinase 1 and 4 (IRAK1/4) complex contributes to adaptive resistance in FLT3-mutant AML cells (Melgar et al. 2019). This result supports our observation about the association of immune response pathways with drug resistance.

The validation of RNA-seq results was performed for ANGPT2 and catalase by Western blotting. *ANGPT2* mRNA was up-regulated, whereas *CAT* mRNA was down-regulated in HCT116 *p53* ^*−/−*^ cells compared to HCT116 *p53* ^*−*+*/*+^ cells. This was confirmed for protein expression. *CAT* is frequently down-regulated in tumors (Glorieux et al. 2014), e.g. Breast cancer was characterized by down-regulation of catalase and concomitant overexpression of SOD (Wang et al. 2017). On the other hand, upregulation of *ANGPT2* was associated with liver metastasis in colon cancer (Urosevic et al. 2020).

Network analysis pointed out “cancer”, “organismal injury and abnormalities” for network 1, “cellular assembly and organization”, “molecular transport” for network 2, “organismal injury and abnormalities”, “carbohydrate metabolism” for network 3 as major affected biological functions. Due to *TP53* deletion, disruption in the DNA repair and apoptosis mechanisms were probably leading to aberrancies in cellular and organismal organization, ultimately increasing tumorigenic and cancer progressive potential. In network 1, the genes encoding histone H4, cyclin A and NFκB possessed the highest number of nodes. CD3 and HSP70 had the highest number of nodes in the center of network 2, implying an influence of cell cycle regulation, inflammation and heat-shock response for drug resistance. Importantly, the appearance of molecular transport genes in network 2 highlighted a possible cross-talk between p53 and cellular transporters to promote the MDR in cancer cells. A member of ABC transporters, the *ABCB1/MDR1* gene, is transcriptionally dependent on p53, where wild type p53 negatively affects *ABCB1/MDR1* gene expression through sequence-specific binding to the downstream promoter (Strauss et al. 1995). On the contrary, mutant p53 activated *ABCB1/MDR1* promoter in different cell lines (Nguyen et al. 1994; Sampath et al. 2001). The *ERK1/2* gene had the highest number of nodes in network 3, pointing out a contribution of ERK-regulated cell proliferation pathway to drug resistance. NFκB resided in the center of the merged network together with PI3K and HSP70, implying a contribution of inflammatory pathways together with cell cycle and heat shock response phenomena in the MDR phenotype. Th1, Th2 pathways and CD28 signaling were among the most affected signaling pathways in HCT116-*p53 *^*−/−*^ cells supporting the hypothesis that inflammatory pathways play an important role in the MDR phenotype.

## Conclusions

In conclusion, the gene expression profiles of HCT116 *p53* ^*−/−*^ and HCT116 *p53* ^+*/*+^ colon cancer cell lines were analyzed by RNA sequencing, mFISH and aCGH, to identify differentially expressed genes, affected protein networks, pathways, biological functions in addition to chromosomal aberrations in a comparative manner. Various genes, pathways and networks were identified that might be associated with drug resistance and aggressive behavior of colon cancer. This study clearly demonstrates that drug resistance in *TP53*-knockout cells is rather determined by multiple than by single factors. It is apparent that multi-factorial drug resistance complicates the development of novel treatment strategies. Nevertheless, our study may represent a starting point to design more specific and promising anti-cancer strategies bypassing drug resistance.

## Supplementary Information

Below is the link to the electronic supplementary material.

Table S1: Differentially expressed genes in HCT116 *p53* ^*−/−*^ cells compared to HCT116 *p53* ^*+/+*^ cells (threshold: ± 7-fold changed expression) (DOCX 34 KB)

Table S2: Full list of all deregulated genes in HCT116 *p53* *−/− *cells involved in resistance mechanisms (XLSX 18 KB)

Table S3: List of deregulated genes in HCT116 *p53* ^*−/−*^ residing at top 10 biological functions (XLSX 11 KB)

## Data Availability

All data generated or analysed during this study are included in this published article.

## Abbreviations

aCGH: Array comparative genomic hybridization
mFISH: Multicolor fluorescence in situ hybridization
MDR: Multidrug resistance
RNA-Seq: RNA sequencing

## References

[CR1] Agarwal ML, Agarwal A, Taylor WR, Stark GR (1995). P53 controls both the G(2)/M and the G(1) cell-cycle checkpoints and mediates reversible growth arrest in human fibroblasts. Proc Natl Acad Sci USA.

[CR2] Aust N, Schule S, Altendorf-Hofmann AK (2013). Loss of chromosome 4 correlates with better long-term survival and lower relapse rate after R0-resection of colorectal liver metastases. J Cancer Res Clin Oncol.

[CR3] Baker SJ, Fearon ER, Nigro JM (1989). Chromosome-17 deletions and p53 gene-mutations in colorectal carcinomas. Science.

[CR4] Bartlett NL, Costello BA, LaPlant BR (2018). Single-agent ibrutinib in relapsed or refractory follicular lymphoma: a phase 2 consortium trial. Blood.

[CR5] Bertheau P, Espie M, Turpin E (2008). TP53 status and response to chemotherapy in breast cancer. Pathobiology.

[CR6] Bhattacharjee RN, Park KS, Okada K (2005). Microarray analysis identifies apoptosis regulatory gene expression in HCT116 cells infected with thermostable direct hemolysin-deletion mutant of *Vibrio parahaemolyticus*. Biochem Biophys Res Commun.

[CR7] Blunt MD, Koehrer S, Dobson RC (2017). The dual Syk/JAK inhibitor cerdulatinib antagonizes B-cell receptor and microenvironmental signaling in chronic lymphocytic leukemia. Clin Cancer Res.

[CR8] Bode AM, Dong ZG (2004). Post-translational modification of p53 in tumorigenesis. Nat Rev Cancer.

[CR9] Brachtendorf S, Wanger RA, Birod K (1863). (2018) Chemosensitivity of human colon cancer cells is influenced by a p53-dependent enhancement of ceramide synthase 5 and induction of autophagy. Biochim Biophys Acta Mol Cell Biol Lipids.

[CR10] Bunz F, Dutriaux A, Lengauer C (1998). Requirement for p53 and p21 to sustain G2 arrest after DNA damage. Science.

[CR11] Bunz F, Hwang PM, Torrance C (1999). Disruption of p53 in human cancer cells alters the responses to therapeutic agents. J Clin Invest.

[CR12] Callahan MJ, Nagymanyoki Z, Bonome T (2008). Increased HLA-DMB expression in the tumor epithelium is associated with increased CTL infiltration and improved prognosis in advanced-stage serous ovarian cancer. Clin Cancer Res.

[CR13] Chang L, Guo F, Wang Y (2014). MicroRNA-200c regulates the sensitivity of chemotherapy of gastric cancer SGC7901/DDP cells by directly targeting RhoE. Pathol Oncol Res.

[CR14] Chen MB, Zhu YQ, Xu JY (2012). Value of TP53 status for predicting response to neoadjuvant chemotherapy in breast cancer: a meta-analysis. PLoS ONE.

[CR15] Chen JW, Huang CQ, Chen KM (2018). Overexpression of LBH is associated with poor prognosis in human hepatocellular carcinoma. Oncotargets Ther.

[CR16] Choudhri P, Rani M, Sangwan RS, Kumar R, Kumar A, Chhokar V (2018). *De novo* sequencing, assembly and characterisation of *Aloe vera* transcriptome and analysis of expression profiles of genes related to saponin and anthraquinone metabolism. BMC Genomics.

[CR17] Coker-Gurkan A, Arisan ED, Obakan P, Palavan-Unsal N (2015). Lack of functional p53 renders DENSpm-induced autophagy and apoptosis in time-dependent manner in colon cancer cells. Amino Acids.

[CR18] Cross RS, Malaterre J, Davenport AJ (2015). Therapeutic DNA vaccination against colorectal cancer by targeting the MYB oncoprotein. Clin Transl Immunol.

[CR19] Dawood M, Hamdoun S, Efferth T (2018). Multifactorial modes of action of arsenic trioxide in cancer cells as analyzed by classical and network pharmacology. Front Pharmacol.

[CR20] Deng HX, Geng QQ, Ji T, Yang AM (2018). miR-539 enhances chemosensitivity to cisplatin in non-small cell lung cancer by targeting DCLK1. Biomed Pharmacother.

[CR21] Dominijanni A, Gmeiner WH (2018). Improved potency of F10 relative to 5-fluorouracil in colorectal cancer cells with p53 mutations. Cancer Drug Resist.

[CR22] Efferth T, Konkimalla VB, Wang YF (2008). Prediction of broad spectrum resistance of tumors towards anticancer drugs. Clin Cancer Res.

[CR23] Fischer M (2017). Census and evaluation of p53 target genes. Oncogene.

[CR24] Funato T, Satou J, Kozawa K, Fujimaki S, Miura T, Kaku M (2001). Use of c-myb antisense oligonucleotides to increase the sensitivity of human colon cancer cells to cisplatin. Oncol Rep.

[CR25] Ge Y, Weygant N, Qu D (2018). Alternative splice variants of DCLK1 mark cancer stem cells, promote self-renewal and drug-resistance, and can be targeted to inhibit tumorigenesis in kidney cancer. Int J Cancer.

[CR26] Glorieux C, Auquier J, Dejeans N (2014). Catalase expression in MCF-7 breast cancer cells is mainly controlled by PI3K/Akt/mTor signaling pathway. Biochem Pharmacol.

[CR27] Gomes AS, Ramos H, Soares J, Saraiva L (2018). p53 and glucose metabolism: an orchestra to be directed in cancer therapy. Pharmacol Res.

[CR28] Green DR, Kroemer G (2009). Cytoplasmic functions of the tumour suppressor p53. Nature.

[CR29] Grommes C, Pastore A, Palaskas N (2017). Ibrutinib unmasks critical role of Bruton tyrosine kinase in primary CNS lymphoma. Cancer Discov.

[CR30] Gunasegaran B, Neilsen PM, Smid SD (2020). P53 activation suppresses irinotecan metabolite SN-38-induced cell damage in non-malignant but not malignant epithelial colonic cells. Toxicol In Vitro.

[CR31] Hainaut P, Soussi T, Shomer B (1997). Database of p53 gene somatic mutations in human tumors and cell lines: updated compilation and future prospects. Nucleic Acids Res.

[CR32] Hernlund E, Ihrlund LS, Khan O (2008). Potentiation of chemotherapeutic drugs by energy metabolism inhibitors 2-deoxyglucose and etomoxir. Int J Cancer.

[CR33] Hientz K, Mohr A, Bhakta-Guha D, Efferth T (2017). The role of p53 in cancer drug resistance and targeted chemotherapy. Oncotarget.

[CR34] Introna M, Golay J (1999). How can oncogenic transcription factors cause cancer: a critical review of the myb story. Leukemia.

[CR35] Janssen A, Schiffmann S, Birod K (2008). p53 is important for the anti-proliferative effect of ibuprofen in colon carcinoma cells. Biochem Biophys Res Commun.

[CR36] Kabir MF, Mohd Ali J, Haji Hashim O (2018). Microarray gene expression profiling in colorectal (HCT116) and hepatocellular (HepG2) carcinoma cell lines treated with *Melicope ptelefolia* leaf extract reveals transcriptome profiles exhibiting anticancer activity. PeerJ.

[CR37] Kadioglu O, Cao J, Kosyakova N, Mrasek K, Liehr T, Efferth T (2016). Genomic and transcriptomic profiling of resistant CEM/ADR-5000 and sensitive CCRF-CEM leukaemia cells for unravelling the full complexity of multi-factorial multidrug resistance. Sci Rep.

[CR38] Kadioglu O, Fu YJ, Wiench B, Zu YG, Efferth T (2016). Synthetic cajanin stilbene acid derivatives inhibit c-MYC in breast cancer cells. Arch Toxicol.

[CR39] Khonthun C, Saikachain N, Popluechai S (2020). Microarray analysis of gene expression involved in butyrate-resistant colorectal carcinoma HCT116 cells. Asian Pac J Cancer Prev.

[CR40] Krais AM, Speksnijder EN, Melis JP (2016). The impact of p53 on DNA damage and metabolic activation of the environmental carcinogen benzo[a]pyrene: effects in Trp53(+/+), Trp53(+/-) and Trp53(-/-) mice. Arch Toxicol.

[CR41] Kralova V, Brigulova K, Cervinka M, Rudolf E (2009). Antiproliferative and cytotoxic effects of sodium selenite in human colon cancer cells. Toxicol In Vitro.

[CR42] Lane DP (1992). Cancer. p53, guardian of the genome. Nature.

[CR43] Li K, Lu Y, Liang J (2009). RhoE enhances multidrug resistance of gastric cancer cells by suppressing Bax. Biochem Biophys Res Commun.

[CR44] Liehr T, Pellestor F, Liehr T (2009). Molecular cytogenetics: the standard FISH and PRINS procedure. Fluorescence *in situ* hybridization (FISH)—application guide.

[CR45] Liehr T, Mrasek K, Kosyakova N, Liehr T (2009). Multiplex FISH and spectral karyotyping. Fluorescence *in situ* hybridization (FISH)—application guide.

[CR46] Liehr T, Mrasek K, Kosyakova N, Liehr T (2009). FISH banding techniques. Fluorescence *in situ* hybridization (FISH)—application guide.

[CR47] Lin ZP, Belcourt MF, Cory JG, Sartorelli AC (2004). Stable suppression of the R2 subunit of ribonucleotide reductase by R2-targeted short interference RNA sensitizes p53(-/-) HCT-116 colon cancer cells to DNA-damaging agents and ribonucleotide reductase inhibitors. J Biol Chem.

[CR48] Liu DP, Song H, Xu Y (2010). A common gain of function of p53 cancer mutants in inducing genetic instability. Oncogene.

[CR49] Ma M, Yang J, Wang B, Zhao Z, Xi JJ (2017). High-throughput identification of miR-596 inducing p53-mediated apoptosis in HeLa and HCT116 cells using cell microarray. SLAS Technol.

[CR50] Makino S, Takahashi H, Okuzaki D (2020). DCLK1 integrates induction of TRIB3, EMT, drug resistance and poor prognosis in colorectal cancer. Carcinogenesis.

[CR51] Martner A, Aydin E, Hellstrand K (2019). NOX2 in autoimmunity, tumor growth and metastasis. J Pathol.

[CR52] Matsuura K, Canfield K, Feng W, Kurokawa M (2016). Metabolic regulation of apoptosis in cancer. Int Rev Cell Mol Biol.

[CR53] Melgar K, Walker MM, Jones LM (2019). Overcoming adaptive therapy resistance in AML by targeting immune response pathways. Sci Transl Med.

[CR54] Millen R, Malaterre J, Cross RS (2016). Immunomodulation by MYB is associated with tumor relapse in patients with early stage colorectal cancer. Oncoimmunology.

[CR55] Mohapatra P, Preet R, Das D (2012). Quinacrine-mediated autophagy and apoptosis in colon cancer cells is through a p53- and p21-dependent mechanism. Oncol Res.

[CR56] Muller PAJ, Vousden KH (2013). p53 mutations in cancer. Nat Cell Biol.

[CR57] Nguyen KT, Liu B, Ueda K, Gottesman MM, Pastan I, Chin KV (1994). Transactivation of the human multidrug resistance (MDR1) gene promoter by p53 mutants. Oncol Res.

[CR58] Ni XF, Zhao LH, Li G (2018). MicroRNA-548-3p and MicroRNA-576-5p enhance the migration and invasion of esophageal squamous cell carcinoma cells via NRIP1 down-regulation. Neoplasma.

[CR59] Oren M (2003). Decision making by p53: life, death and cancer. Cell Death Differ.

[CR60] Pelengaris S, Khan M, Evan G (2002). c-MYC: more than just a matter of life and death. Nat Rev Cancer.

[CR61] Piovesan B, Pennell N, Berinstein NL (1998). Human lymphoblastoid cell lines expressing mutant p53 exhibit decreased sensitivity to cisplatin-induced cytotoxicity. Oncogene.

[CR62] Qu D, Weygant N, Yao J (2019). Overexpression of DCLK1-AL increases tumor cell invasion, drug resistance, and KRAS activation and can be targeted to inhibit tumorigenesis in pancreatic cancer. J Oncol.

[CR63] Ramsay RG, Gonda TJ (2008). MYB function in normal and cancer cells. Nat Rev Cancer.

[CR64] Reed JC (1995). Bcl-2 family proteins: regulators of chemoresistance in cancer. Toxicol Lett.

[CR65] Riley T, Sontag E, Chen P, Levine A (2008). Transcriptional control of human p53-regulated genes. Nat Rev Mol Cell Bio.

[CR66] Saeed M, Jacob S, Sandjo LP (2015). Cytotoxicity of the sesquiterpene lactones neoambrosin and damsin from *Ambrosia maritima* against multidrug-resistant cancer cells. Front Pharmacol.

[CR67] Sampath J, Sun D, Kidd VJ (2001). Mutant p53 cooperates with ETS and selectively up-regulates human MDR1 not MRP1. J Biol Chem.

[CR68] Schultz J, Lorenz P, Ibrahim SM, Kundt G, Gross G, Kunz M (2009). The functional-443T/C osteopontin promoter polymorphism influences osteopontin gene expression in melanoma cells via binding of c-Myb transcription factor. Mol Carcinogen.

[CR69] Sonnemann J, Marx C, Becker S (2014). p53-dependent and p53-independent anticancer effects of different histone deacetylase inhibitors. Br J Cancer.

[CR70] Strauss BE, Shivakumar C, Deb SP, Deb S, Haas M (1995). The MDR1 downstream promoter contains sequence-specific binding sites for wild-type p53. Biochem Biophys Res Commun.

[CR71] Terranova-Barberio M, Pecori B, Roca MS (2017). Synergistic antitumor interaction between valproic acid, capecitabine and radiotherapy in colorectal cancer: critical role of p53. J Exp Clin Cancer Res.

[CR72] Urosevic J, Blasco MT, Llorente A (2020). ERK1/2 Signaling induces upregulation of ANGPT2 and CXCR4 to mediate liver metastasis in colon cancer. Cancer Res.

[CR73] Wang F, Liu X, Yang P (2014). Loss of TACSTD2 contributed to squamous cell carcinoma progression through attenuating TAp63-dependent apoptosis. Cell Death Dis.

[CR74] Wang L, Luo X, Li C (2017). Triethylenetetramine synergizes with pharmacologic ascorbic acid in hydrogen peroxide mediated selective toxicity to breast cancer cell. Oxid Med Cell Longev.

[CR75] Wesolowski S, Birtwistle MR, Rempala GA (2013). A comparison of methods for RNA-seq differential expression analysis and a new empirical Bayes approach. Biosensors (Basel).

[CR76] Wohak LE, Baranski AC, Krais AM, Schmeiser HH, Phillips DH, Arlt VM (2018). The impact of p53 function on the metabolic activation of the carcinogenic air pollutant 3-nitrobenzanthrone and its metabolites 3-aminobenzanthrone and N-hydroxy-3-aminobenzanthrone in human cells. Mutagenesis.

[CR77] Wu C, de Miranda NF, Chen L (2016). Genetic heterogeneity in primary and relapsed mantle cell lymphomas: Impact of recurrent CARD11 mutations. Oncotarget.

[CR78] Zhang JX, Chen ZH, Chen DL (2018). LINC01410-miR-532-NCF2-NF-kappa B feedback loop promotes gastric cancer angiogenesis and metastasis. Oncogene.

[CR79] Zhou JF, Yang JJ, Li K (2013). RhoE is associated with relapse and prognosis of patients with colorectal cancer. Ann Surg Oncol.

