# Supplementary material for: Identification of potential novel drug resistance mechanisms by genomic and transcriptomic profiling of colon cancer cells with p53 deletion
Source: Arch Toxicol. 2021 Jan 30;95(3):959–74. doi: 10.1007/s00204-021-02979-4 (PMC7904745; doi:10.1007/s00204-021-02979-4)
Supplement: Supplementary file 1 — Table S1: Differentially expressed genes in HCT116 p53 −/− cells compared to HCT116 p53 +/+ cells (threshold: ± 7-fold changed expression) (DOCX 34 KB) [file 204_2021_2979_MOESM1_ESM.docx]

**Supplementary Table 1:** Differentially expressed genes in HCT116 p53^(-/-)^ cells compared to HCT116 p53^(+/+)^ cells (threshold: ± 7 fold changed expression)

| Gene | Differential expression (fold change) |
| --- | --- |
| *RND3* | 235.6326561 |
| *MCPH1* | 85.70061021 |
| *DCLK1* | 60.19254063 |
| *ZNF772* | 37.03900828 |
| *ANKRD31* | 37.02760356 |
| *ZNF419* | 32.42012428 |
| *LBH* | 31.9544066 |
| *MYB* | 28.85496296 |
| *MIR4477B* | 26.76770997 |
| *MST1* | 25.17731144 |
| *EID3* | 23.78670336 |
| *ZNF736* | 21.83834998 |
| *MCPH1* | 20.62846166 |
| *CD4* | 20.43138025 |
| *TBX1* | 20.0667324 |
| *KRT81,KRT83,KRT85* | 18.75022145 |
| *RERG* | 18.49732223 |
| *MORC2-AS1* | 18.48899077 |
| *TPI1P2* | 18.48661735 |
| *ACAP1* | 18.44314606 |
| *OR52D1* | 18.44291363 |
| *TNFSF12-TNFSF13* | 18.41641395 |
| *MCPH1* | 18.22908698 |
| *SV2C* | 16.93331413 |
| *FOXL2NB* | 16.82503962 |
| *LINC00921* | 16.8128064 |
| *MLLT11* | 16.80856876 |
| *RIBC2* | 16.78768286 |
| *CYSRT1* | 16.7823874 |
| *NDRG4* | 16.74825408 |
| *LOC101927418* | 16.65237094 |
| *HM13-AS1* | 16.61527475 |
| *IMMT* | 16.6064747 |
| *TCAM1P* | 15.9799502 |
| *LRRC26* | 15.93749549 |
| *SPDYE3* | 15.13807644 |
| *NCF2* | 15.13082331 |
| *EDN1* | 15.1267988 |
| *LOC100132352* | 15.11730702 |
| *RNU6-28P* | 15.08841014 |
| *CDC45* | 14.78052112 |
| *PDCD4-AS1* | 13.44476296 |
| *LOC101929099* | 13.44115765 |
| *FNDC4* | 13.43802659 |
| *PROZ* | 13.43555493 |
| *OR7E14P* | 13.41896492 |
| *FBXL19-AS1* | 13.37552478 |
| *ARL14* | 12.58642311 |
| *NID2* | 12.58080125 |
| *KRTAP5-1* | 12.53933189 |
| *LINC01136* | 12.46755026 |
| *MANEA-AS1* | 12.35632836 |
| *TECPR2* | 12.23439042 |
| *FOXP4-AS1* | 12.18280359 |
| *RALY-AS1* | 12.0985629 |
| *HEPH* | 11.77777778 |
| *TSLP* | 11.76887841 |
| *USP27X-AS1* | 11.76750983 |
| *CBX3P2* | 11.7521507 |
| *PYCARD-AS1* | 11.73822769 |
| *FTH1P3* | 11.72861856 |
| *ISM1* | 11.20975774 |
| *XRCC2* | 11.19986687 |
| *SPIB* | 11.13133309 |
| *LINC00886* | 11.10200281 |
| *LINC00638* | 10.92896866 |
| *FRAS1* | 10.63810325 |
| *CCAT1* | 10.61879405 |
| *PRPS1* | 10.59847458 |
| *ZMAT1* | 10.48502003 |
| *RPE65* | 10.16404827 |
| *LOC648987* | 10.16295159 |
| *STRA6* | 10.0962666 |
| *CABP7* | 10.0910679 |
| *ANGPTL3* | 10.09091637 |
| *TYRP1* | 10.09053437 |
| *LOC642776* | 10.08456151 |
| *MDH1B* | 10.08147575 |
| *LINC00634* | 10.07514479 |
| *MCPH1* | 10.01132155 |
| *STIP1* | 10.01087719 |
| *TENM3* | 9.655296606 |
| *MRPL23-AS1* | 9.407608647 |
| *VDAC2* | 9.34040264 |
| *LOC344887* | 9.24480294 |
| *GRM2* | 9.202579875 |
| *C9orf41-AS1* | 9.154073467 |
| *MCPH1* | 8.934144811 |
| *ANGPT2* | 8.732242134 |
| *NRG1* | 8.622515695 |
| *ZFPM2* | 8.413353105 |
| *ITGB2* | 8.411300889 |
| *RTN1* | 8.410955806 |
| *LINC00514* | 8.410175719 |
| *TFPI2* | 8.409596155 |
| *LOC101929199* | 8.408396817 |
| *LOC339874* | 8.402598745 |
| *C2orf48* | 8.401806539 |
| *KLRG1* | 8.40041144 |
| *ACTG1P17* | 8.398467885 |
| *DUXAP10,LINC01296* | 8.393847376 |
| *CASP4* | 8.390825113 |
| *CDKL1* | 8.374244923 |
| *MCPH1* | 8.331881694 |
| *MCPH1* | 8.327296988 |
| *HIST1H2AM* | 8.306848596 |
| *GINS2* | 8.192583689 |
| *GALNT7* | 8.178197578 |
| *RAD9B* | 8.126829385 |
| *HOXC8* | 8.125148595 |
| *S100A2* | 8.066321993 |
| *PA2G4P4* | 7.987766081 |
| *ABHD16B* | 7.920245619 |
| *DHFR* | 7.916280256 |
| *ZNF594* | 7.882567124 |
| *SNED1* | 7.829813178 |
| *LOC100996579* | 7.74976679 |
| *BRIP1* | 7.711484048 |
| *LOC100287042* | 7.68672695 |
| *LOC100288748* | 7.657449364 |
| *LOC389602* | 7.629217042 |
| *CX3CR1* | 7.57083701 |
| *TUBB1* | 7.569956524 |
| *METTL7A* | 7.56966943 |
| *PRDM12* | 7.566050374 |
| *MAN1B1-AS1* | 7.561291937 |
| *KRT14* | 7.558403032 |
| *LINC00941* | 7.533849859 |
| *ZBED2* | 7.424117447 |
| *HOXA2* | 7.280244754 |
| *LOC100129216* | 7.273911058 |
| *KIAA0101* | 7.261971156 |
| *MCM4* | 7.233937014 |
| *FAM216A* | 7.148220301 |
| *ESRG* | 7.081983587 |
| *LINC00342* | 7.033155171 |
| *DPEP1* | -431.8584535 |
| *ICAM1* | -166.4634468 |
| *EHF* | -163.2817742 |
| *TACSTD2* | -110.0729147 |
| *LAMA4* | -104.720601 |
| *NPM2* | -85.41465767 |
| *NRIP1* | -81.51951502 |
| *HLA-DMB* | -69.67764629 |
| *HCG4B* | -61.25492892 |
| *MIR564,TMEM42* | -58.38036864 |
| *CPM* | -56.5244092 |
| *PRKAA2* | -54.06565597 |
| *FGF17* | -52.65411382 |
| *MSH4,RABGGTB,SNORD45C* | -51.52915282 |
| *ZNF234* | -42.0505542 |
| *ZFP30* | -40.41091708 |
| *P4HA3* | -35.63239219 |
| *ACSL6* | -34.98727399 |
| *SLC22A17* | -34.32403534 |
| *WNT11* | -32.13297102 |
| *CCBE1* | -30.30086305 |
| *FEZ1* | -29.69925981 |
| *CD33* | -29.31257245 |
| *TMTC1* | -27.48032209 |
| *PI15* | -27.32903036 |
| *ZNF850* | -27.00034723 |
| *MIAT,MIATNB* | -25.62749689 |
| *NELL2* | -24.49027912 |
| *TGM2* | -24.37052199 |
| *SLC6A20* | -23.86545682 |
| *LOC730102* | -23.09041945 |
| *FAM184A* | -21.98889993 |
| *KLRC4,KLRC4-KLRK1,KLRK1* | -21.13288322 |
| *CGNL1* | -20.95965395 |
| *AFF3* | -19.04034822 |
| *SERPINA5* | -18.44052205 |
| *SULT2B1* | -18.2085718 |
| *PXDN* | -17.89508001 |
| *CA14* | -17.83061582 |
| *MAT1A* | -17.2398407 |
| *EFHD1* | -17.1984651 |
| *LPPR4* | -17.18807946 |
| *GPR176* | -16.74853685 |
| *CLIC5* | -16.45563802 |
| *DNAH2* | -16.25667381 |
| *ARHGEF25* | -15.75064132 |
| *ABI3BP* | -15.17010878 |
| *RTEL1,RTEL1-TNFRSF6B,TNFRSF6B* | -14.9454243 |
| *TMEM211* | -14.89567464 |
| *ABI3BP* | -14.87772952 |
| *ALX4* | -14.87599653 |
| *PADI2* | -14.55460147 |
| *HIST1H3E* | -14.52251971 |
| *EPGN* | -13.42522806 |
| *LOC100132057* | -13.23659083 |
| *NCAM1* | -13.20791077 |
| *HAPLN2* | -13.13994704 |
| *MGAT3* | -13.07858756 |
| *FBLN2* | -13.07714177 |
| *SSBP3-AS1* | -12.48886674 |
| *KCNN1* | -12.48857356 |
| *BVES* | -12.26165285 |
| *CCDC63* | -12.12639621 |
| *COX6B2* | -12.05921776 |
| *NBPF20* | -11.9119155 |
| *KCNJ5* | -11.89220103 |
| *PAOX* | -11.63791875 |
| *BMP6* | -11.59372222 |
| *TMEM158* | -11.45584802 |
| *LHB,LOC101059948* | -11.42608688 |
| *COL26A1* | -11.30880637 |
| *LIMS2* | -11.2986649 |
| *KLHL30* | -11.29393819 |
| *FAM71E1* | -11.15987752 |
| *ULK2* | -10.99928747 |
| *PCOLCE-AS1* | -10.73629189 |
| *PTGER2* | -10.70582627 |
| *LOC100129055* | -10.7037075 |
| *ZNF599* | -10.70184683 |
| *SH3TC1* | -10.64125127 |
| *CTSH* | -10.63494959 |
| *CPVL* | -10.53551078 |
| *C1S* | -10.48705824 |
| *FRMD4B* | -10.48039998 |
| *ZNF391* | -10.40286433 |
| *PROM2* | -10.33895717 |
| *BCL11A* | -10.27597946 |
| *CCDC183-AS1* | -10.17047553 |
| *BDKRB1* | -10.12952259 |
| *BCAR3* | -9.942080662 |
| *FOLR1* | -9.633552699 |
| *BDKRB2* | -9.574910422 |
| *SPSB4* | -9.513708351 |
| *CPXM2* | -9.51055197 |
| *SCARA5* | -9.509419903 |
| *FGD5* | -9.506541006 |
| *SORCS2* | -9.506393538 |
| *CPQ* | -9.316922068 |
| *KCNK9* | -9.169532342 |
| *HOXA11-AS* | -8.949060458 |
| *TSKS* | -8.927819939 |
| *NLRP14,RBMXL2* | -8.922145367 |
| *ENKD1* | -8.920916474 |
| *CABP4* | -8.86941354 |
| *ALOX5* | -8.851999028 |
| *RASSF4* | -8.772742834 |
| *RBM46* | -8.721634359 |
| *AMBP* | -8.624156247 |
| *RAB27B* | -8.536198478 |
| *LOC102724539* | -8.423697669 |
| *MIR548F1* | -8.414665798 |
| *CYTH4* | -8.413164459 |
| *FAM20A* | -8.363926315 |
| *MIR7641-2* | -8.339466326 |
| *DYNC2H1* | -8.314981586 |
| *CASS4* | -8.212568807 |
| *PARK2* | -8.148651953 |
| *MB* | -8.093345972 |
| *KCNJ1* | -8.084557797 |
| *VASH2* | -8.055892834 |
| *CORO1A,LOC606724* | -8.019001281 |
| *MAMDC2* | -7.9941508 |
| *PIWIL2* | -7.924109575 |
| *LOC101926975* | -7.883707228 |
| *GNG12-AS1* | -7.872919016 |
| *SLC26A4* | -7.868327402 |
| *PLTP* | -7.852396125 |
| *ANXA2P2* | -7.805149208 |
| *DHX58* | -7.800978102 |
| *MKLN1* | -7.752263701 |
| *LOC388242* | -7.742320244 |
| *MNS1* | -7.735278681 |
| *GUCY2D* | -7.727579591 |
| *ADAMTS15* | -7.724297549 |
| *NFATC4* | -7.618997522 |
| *FBXO24* | -7.587930478 |
| *CLDN2* | -7.553721051 |
| *C10orf25* | -7.546165076 |
| *LINC01089* | -7.493329624 |
| *MAMDC2* | -7.485388066 |
| *FAM20A* | -7.453430692 |
| *TUBAL3* | -7.43164861 |
| *CARD11* | -7.428761242 |
| *NPHP3-ACAD11* | -7.389385525 |
| *WNT8B* | -7.378497562 |
| *DRC7* | -7.369726777 |
| *HLA-DRB1* | -7.352802826 |
| *EGR4* | -7.336068397 |
| *NPR3* | -7.329464517 |
| *IL18R1* | -7.250350451 |
| *ARHGAP31* | -7.209893699 |
| *SH3BP5* | -7.206634126 |
| *HNRNPUL1* | -7.170499796 |
| *GRHL3* | -7.152525972 |
| *SAMD14* | -7.135033878 |
| *ACVRL1* | -7.134072594 |
| *ADRA2B* | -7.133823638 |
| *IRF4* | -7.133224202 |
| *APBA1* | -7.129394868 |
| *GRIP2* | -7.120315437 |
| *MUC4* | -7.067438476 |
| *CATSPERG* | -7.063860652 |
| *CST6* | -7.062355862 |
|  |  |
